# Elevated iron levels in Alzheimer’s disease brain was partially due to increased cytosolic expression of hemoglobin in neurons

**DOI:** 10.1002/alz.090319

**Published:** 2025-01-03

**Authors:** Blaine Russell Roberts, Soumya Mukherjee, Anne M Roberts, Madison E Platt, Catriona MacLean, Ashley I. Bush, Ian Birchall, Colin L. Masters

**Affiliations:** ^1^ Emory University School of Medicine, Atlanta, GA USA; ^2^ Washington University School of Medicine, St. Louis, MO USA; ^3^ Emory School of Medicine, Atlanta, GA USA; ^4^ Howard Florey Institute, Melbourne Australia; ^5^ The Florey Institute of Neuroscience and Mental Health, The University of Melbourne, Australia, Melbourne, VIC Australia; ^6^ The Florey Institute of Neuroscience and Mental Health, PARKVILLE, VIC Australia; ^7^ Florey Institute of Neuroscience and Mental Health, University of Melbourne, Parkville, VIC Australia

## Abstract

**Background:**

A better understanding of the molecular process that drive Alzheimer’s disease(AD) are required to develop effective biomarkers and therapies. This includes determining how essential elements like Fe, Cu and Zn are involved in the disease. In the literature there is debate over the role of iron in AD and there are reports of increased, decreased and unchanged levels of Fe in AD brain. Most of the studies have use a standard bulk analysis of Fe in brain tissue. However, this does not yield the necessary molecular information required to determine how the metalloenzymes that utilize Fe are affected in the disease.

**Method:**

We used a combination of biochemical fraction and size exclusion inductively coupled plasma mass spectrometry to conduct a metalloproteomics analysis of post mortem human brain tissue from control and Alzheimer’s Disease cases.

**Result:**

We observed a significant increase in a single iron containing protein. We used bottom up proteomics and purification to determine the protein was hemoglobin. We further confirmed increase expression of hemoglobin and the binding partner haptoglobin in neurons using immunoshitochemistry. We also use RNAscope to show that neurons express the MRNA for both hemoglobin and haptoglobin which was surprising as these are classic blood proteins.

**Conclusion:**

These results clarify that changes in iron proteins do occur in AD brain and open up a new area of research to understand the role of hemoglobin and haptoglobin in neuronal function.

